# Dynamics in the O(2 × 1) adlayer on Ru(0001): bridging timescales from milliseconds to minutes by scanning tunneling microscopy†

**DOI:** 10.1039/d2cp02363f

**Published:** 2022-06-13

**Authors:** Leonard Gura, Zechao Yang, Joachim Paier, Florian Kalaß, Matthias Brinker, Heinz Junkes, Markus Heyde, Hans-Joachim Freund

**Affiliations:** Fritz-Haber-Institut der Max-Planck-Gesellschaft Faradayweg 4-6 14195 Berlin Germany heyde@fhi-berlin.mpg.de +49 30 8413 4149; Humboldt Universität zu Berlin Unter den Linden 6 10099 Berlin Germany

## Abstract

The dynamics within an O(2 × 1) adlayer on Ru(0001) is studied by density functional theory and high-speed scanning tunneling microscopy. Transition state theory proposes dynamic oxygen species in the reduced O(2 × 1) layer at room temperature. Collective diffusion processes can result in structural reorientations of characteristic stripe patterns. Spiral high-speed scanning tunneling microscopy measurements reveal this reorientation as a function of time in real space. Measurements, ranging over several minutes with constantly high frame rates of 20 Hz resolved the gradual reorientation. Moreover, reversible fast flipping events of stripe patterns are observed. These measurements relate the observations of long-term atomic rearrangements and their underlying fast processes captured within several tens of milliseconds.

## Abstract 2

The dynamics within an O(2 × 1) adlayer on Ru(0001) is studied by density functional theory and high-speed scanning tunneling microscopy.

## Graphical abstract

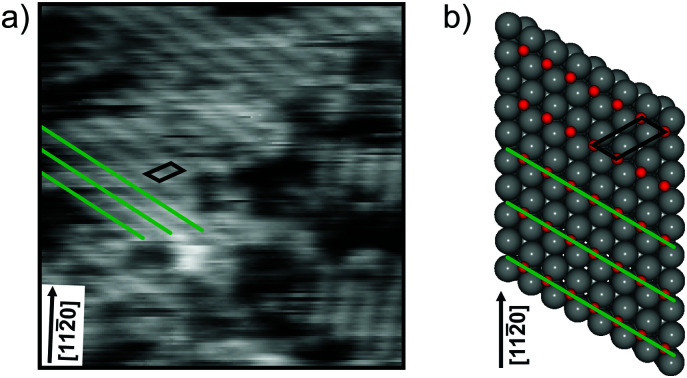

## 1 Introduction

In numerous research fields, scanning tunneling microscopy (STM) is a popular surface science tool. Since its invention it has been used to reveal numerous surface structures^1–3^ and properties.^4,5^ The focus in chemical physics, especially in model catalyst systems is shifting towards the study of dynamic systems, such as mobile adsorbates on surfaces.^6–15^ To capture the dynamics on short timescales, the frame rate of STMs has to be increased.^16^ Therefore, the way how STM measurements are performed needs to be changed considerably.^17^

In the present study we apply a custom built high-speed STM using an innovative spiral scan pattern to investigate the well studied sample system of chemisorbed oxygen on Ruthenium, which has been shown to be important for model catalyst preparation and thin film growth. The oxygen covered Ru(0001) surface is investigated at room temperature. Molecular oxygen dissociates on the Ru surface and forms various superstructures depending on the sample temperature. Close to room temperature, the O(2 × 1) superstructure is stable.^18–20^ LEED studies were performed that showed the spontaneous formation of the O(2 × 1) structure if the coverage exceeds 0.37 ML.^21^ Using STM a characteristic stripe pattern of the O(2 × 1) phase without showing individual atomic sites was revealed. While within the stripe pattern, individual oxygen vacancies were resolved.^19^ In the literature, temperature and coverage dependent studies have been performed, showing individual static snapshots of the adlayer configuration. On the basis of those results, possible formation processes of the O(2 × 1) structure were discussed^20^ including 1D growth of individual line segments and 2D ordering by forming line pairs.^19^ To the best of our knowledge, so far, the dynamics inside the O(2 × 1) layer and the dynamics to form the layer have not been resolved in real space.

We provide real time and real space measurements that reveal vacancy diffusion inside the O(2 × 1) layer. Fast flipping events could be captured within several tens of milliseconds, while rearrangements of the stripe patterns on the nanometer scale are observed within several minutes while scanning continuously with 20 frames s^−1^. Scanning at this constantly high frame rate allows to analyze the connection between slow large scale phenomena and very fast single elementary processes.

## 2 Theoretical modeling and experimental details

To examine the potential energy surface of migrating oxygen in the O(2 × 1) adlayer on Ru(0001) involving O vacancies, *i.e.* a slightly reduced adlayer, local minima and connecting transition structures were optimized based on DFT. The Kohn–Sham–Schrödinger equations were solved within the projector-augmented wave (PAW)^22,23^ approach to describe the interaction between valence electrons and ionic cores as implemented in the Vienna *ab initio* simulation package, VASP.^24,25^ The PAW pseudopotential for Ru was generated based on the electron configuration 4s^2^ 4p^6^ 4d^8^, and the corresponding electron configuration for O was generated *via* a 2s^2^ 2p^4^ configuration (PAW datasets as of version 5.4). As described in detail elsewhere,^26^ we employed six Ru layers in periodic slab models together with 10 Å of a vacuum gap in direction normal to the surface. The (4 × 2) supercell calculations use (3 × 6) Monkhorst-Pack^27^*k*-point meshes to sample Brillouin zones. The Methfessel-Paxton method^28^ for numerical integrations over reciprocal space was used together with a (finite temperature) smearing width of 0.1 eV. A plane wave kinetic energy cutoff of 600 eV was applied. Electronic and ionic optimizations were stopped using break criteria of 10^−5^ eV and 0.01 eV Å^−1^, respectively. Transition structures were localized by virtue of the improved dimer method^29,30^ deploying a break criterion of 0.04 eV Å^−1^. After successful optimization and subsequent frequency calculation to proof the structure a first order saddle point, slab models were reoptimized excluding the migrating oxygen in bridge position and the two directly bound Ru atoms. The coordinates of the three bottommost Ru layers were also kept frozen in (ideal) bulk positions.

To describe the interelectron exchange and correlation effects, the *meta*-generalized-gradient-approximation (*meta*-GGA) SCAN^31^ was invoked. This acronym stands for strongly constrained and appropriately normed. We had applied this functional in previous work and compared its performance with the supposedly more accurate random-phase approximation (RPA).^26^ SCAN energy barriers are estimated to be 0.139 eV higher than corresponding RPA results.

Experimental measurements were performed with the custom built STM, especially designed for high-speed acquisition.^32–34^ The conventional slow raster scan uses commercial hardware and software, while for the high-speed measurements self programmed electronics and voltage input signals are applied.^32,33^ In the present study, we scanned with Archimedean spirals and at constant angular velocity (CAV). For visualization, the image contrast is enhanced by using histogram equalization with kernel sizes of 1/4 of the image size. The images are slightly smoothened with Gaussian filters. Sample preparation and measurements were performed in ultra-high vacuum (UHV) at a base pressure of 10^−10^ mbar. The atomically flat Ru(0001) surface was prepared by repeating cycles of Ar^+^ bombardment, annealing in UHV at 1300 K for up to 5 min, and annealing in oxygen atmosphere for 20 min. After the Ru crystal cooled down below 380 K, molecular oxygen was exposed at 1–2 × 10^−6^ mbar for 1–2 min. All STM measurements are performed at 300 K.

## 3 Results and discussion

**Fig. 1:**
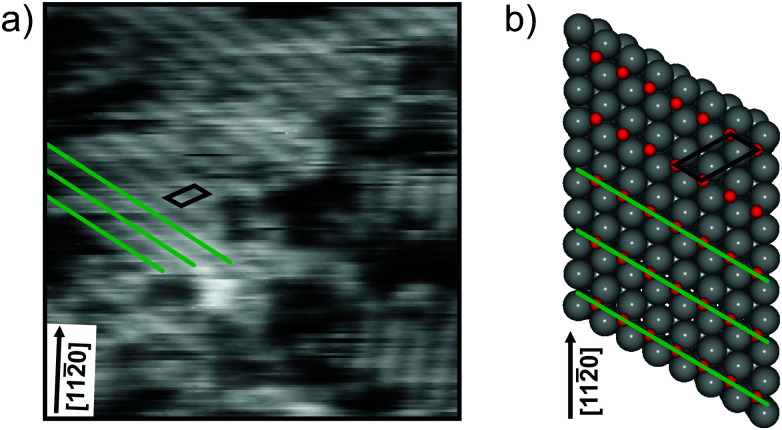
O(2 × 1) adlayer on Ru(0001). (a) Constant current STM image with resolved line features characteristic for the O(2 × 1) phase (*V*_S_ = 0.6 V, *I*_T_ = 1.4 nA, scan area = 9 × 9 nm^2^, acquisition time = 100 s). The straight lines are marked in green and the unit cell is drawn in black. For comparison, the atomic model is shown in (b).

To generate an overview image of the O(2 × 1) structure on the flat Ruthenium surface, we performed STM measurements using the conventional raster scan in constant current mode. The STM image in Fig. 1a was acquired within 100 s and shows the characteristic stripe pattern of the O(2 × 1) structure that has been reported in the literature.^19,20,35^ The single stripes are highlighted with green lines. In the right section of the STM image, a domain with different stripe orientation is detected. In larger overview images, several domains of different orientations rotated by 120 degrees can be identified, as reported in the literature.^20,35^ In the first part of this paper, we focus on a single domain with one stripe orientation. The corresponding unit cell is drawn in black in Fig. 1. For visualization, the schematic model of the adsorbed O atoms on Ruthenium is shown in Fig. 1b.

From the slow scan STM image in Fig. 1a, it is obvious that the O(2 × 1) layer is not completely closed. Depleted areas next to the stripe pattern and single depleted regions along the 1D lines are detectable. This observation is in agreement with reported vacancies in the O(2 × 1) layer.^19^ In a recent publication, we resolved dynamic hopping events in an O(2 × 2) layer and could show the relatively high mobility of oxygen vacancies within the adlayer at room temperature.^26^ The question arises whether or not the vacancies in the O(2 × 1) layer are also mobile.

**Fig. 2:**
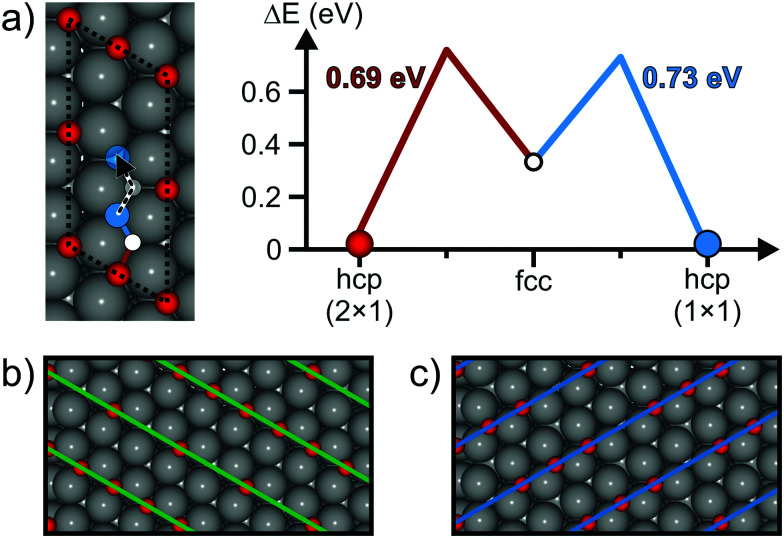
Possible diffusion pathway of oxygen in the 3O(4 × 2) structure on Ru(0001). (a) Unit cell for theoretical calculations with color coded sites along the oxygen diffusion pathway. Energy differences for occupied sites along the diffusion pathway calculated with SCAN are plotted in the right diagram. (b) Sections of repeated unit cells of the initial 3O(4 × 4) structure (2.3 m × 1.3 nm). (c) Section of repeated unit cells after oxygen diffusion from the hcp (2 × 1) site to the hcp (1 × 1) site.

To answer the question whether oxygen diffusion at room temperature is feasible, we performed DFT calculations. SCAN calculations are performed in analogy to ref. 26, where we showed that the energy differences determined with SCAN are similar to free energy barriers. For DFT calculations, the vacancy in the O(2 × 1) layer is modeled by the 3O(4 × 2) structure. The unit cell is marked with a dotted line in Fig. 2a and the proposed diffusion path of the oxygen atom towards the vacant site is marked with color coded straight lines. The diagram shows the corresponding energy differences for single diffusion steps. The barriers for the diffusion *via* the fcc site to the adjacent vacant hcp site on the (1 × 1) grid are in the range of 0.69 eV to 0.73 eV. At room temperature, these energy differences result in jump rates in the order of 3 to 16 Hz, according to Eyring's transition state theory.^36^ Remarkably, the occupation of the hcp (1 × 1) site is energetically more favorable by 0.07 eV. This energy difference can be explained with the minimum repulsion of oxygen atoms at this Fermat point.

We found that the diffusion of oxygen towards the next adjacent vacant hcp site on the (2 × 1) grid in the center of the unit cell is not feasible. The fcc site along the dotted path is very unstable, which may even result in oxygen desorption.

Considering the diffusion event towards the hcp (1 × 1) site in several unit cells leads to Fig. 2b and c. Fig. 2b shows the 3O(4 × 2) structure in the same orientation as the unit cell drawn in Fig. 2a. The orientation of the stripe pattern is marked with green lines. Due to the periodic vacancies, every second stripe is less dense in terms of oxygen concentration. After the diffusion of oxygen to the hcp (1 × 1) sites, the orientation of the stripes changes by 120 degrees (blue lines). This reorientation indicates that at high local oxygen vacancy concentrations in the O(2 × 1) layer the orientation of stripe patterns can change upon diffusion processes. The question arises whether or not these diffusion processes that lead to structural changes on larger length scales can also be resolved in real space and in real time.

**Fig. 3:**
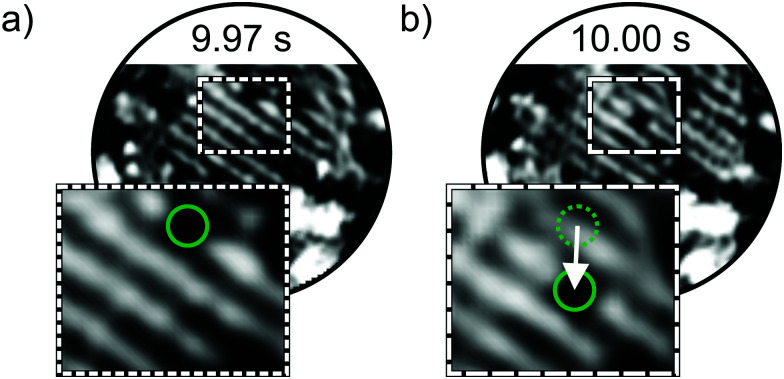
High-speed spiral STM images of the O(2 × 1) adlayer on Ru(0001). (a) and (b) are consecutive spiral STM images recorded with 30 frames s^−1^. An oxygen vacancy appearing as depletion changes its position from (a) to (b). The vacancy is marked with a green circle in the zoomed insets. The dotted green circle in (b) marks the previous vacancy position from (a). (*V*_S_ = 0.6 V, *I*_T_ = 1.4 nA, scan diameter = 9 nm, acquisition time = 33 ms, scan dimensions of insets: 2.7 × 2.2 nm^2^).

To resolve the atom dynamics, the frame rate of the STM measurements must be sufficiently high. By applying the spiral high-speed scan to the same area as shown in Fig. 1a, STM images could be acquired within 33 ms. Fig. 3a and b show two consecutive spiral STM images. The stripe pattern is resolved and comparable to the slow raster scan. The regions marked with dashed rectangles in Fig. 3 magnify the parallel arranged 1D lines. Along the upper 1D line segment a depletion is detected. The contrast is similar to the one described in ref. 19 and the depletion is attributed to an oxygen vacancy. In the consecutive frame 33 ms later, the vacancy jumps to an adjacent position as indicated by the white arrow in Fig. 3b. More of these hopping events in the O(2 × 1) layer could be resolved. The oxygen vacancies show jump rates in the order of 0.1 to 1 Hz. In the ESI,† we provide the frame series before and after the hopping event shown in Fig. 3 in the form of a slow-motion video.

**Fig. 4:**
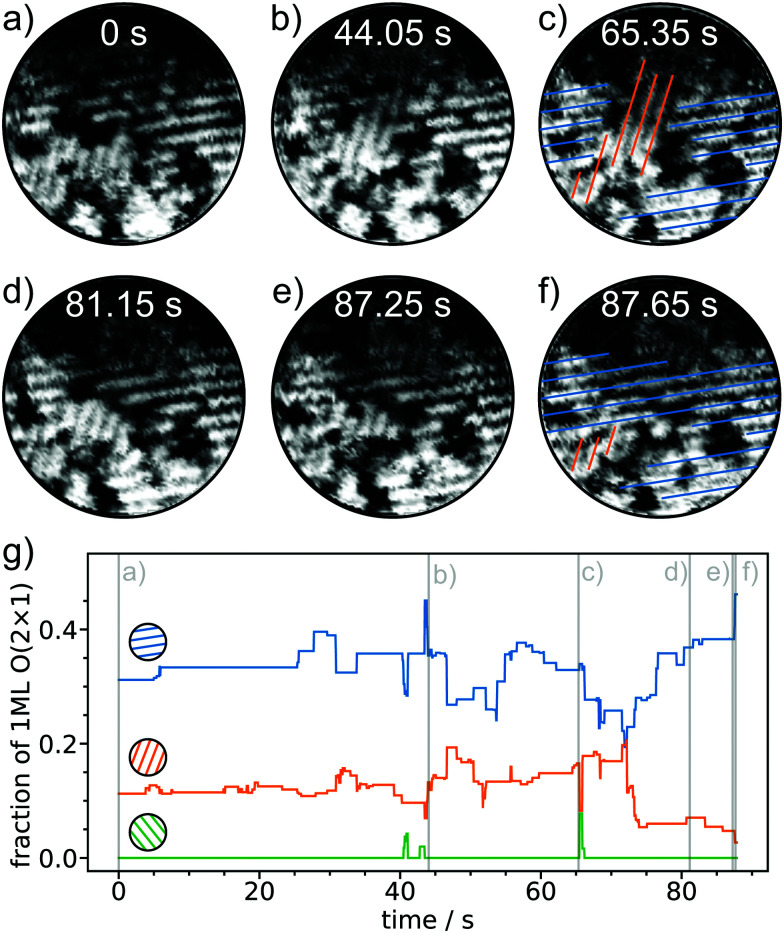
Structural evolution in the O(2 × 1) phase over time. Six STM images are shown that are extracted from a longer image series acquired with 20 frames s^−1^ (*V*_S_ = 1 V, *I*_T_ = 1 nA, scan diameter = 9 nm, acquisition time per image = 50 ms). The orientations of the 1D stripes are marked with colored lines in (c) and (f). (g) Evolution of the normalized length of 1D lines with different orientations. The time of the images in (a)–(f) are marked with vertical gray lines.

The atomic species in a single domain of the O(2 × 1) adlayer are mobile on the Ru(0001) surface. Now we focus on areas with differently oriented domains and analyze scan sequences that are extracted from longer measurements acquired with constantly high frame rates of 20 frames s^−1^. In Fig. 4, six spiral STM images are shown covering almost 1.5 min. Fig. 4g plots the normalized length of the 1D stripes of the respective domains. The provided color code refers to the stripe orientations. In Fig. 4a, two different orientations can be identified: one domain with more horizontal 1D lines (blue) in the right section of the image and more vertical lines (orange) in the lower part of the image. With time the vertical stripes (orange lines) grow and cross the horizontal (blue) lines after roughly 65 seconds in Fig. 4c. Fig. 4g shows that the orange domain grows at the expense of the blue domain. Later, this process of the growing orange domain is reverted. Again, the blue and the orange line in Fig. 4g are opposed. Within the 87 seconds of the scan, the orange domain increases and decreases in size several times until the final configuration is dominated by the blue domain. 22 seconds after the configuration with the orange domain in the center in Fig. 4c, the blue domain dominates the central area in Fig. 4f. In comparison, Fig. 4c and f show a similar structural rearrangement as proposed in the theoretical model shown in Fig. 2b and c. In contrast to the collective jumps in the DFT model due to the periodic boundary conditions, the rearrangement shown in Fig. 4 is a result of numerous dynamic processes at the atomic scale. To show the complete STM image sequence from which Fig. 4 was extracted, we provide a real time video in the ESI.†

**Fig. 5:**
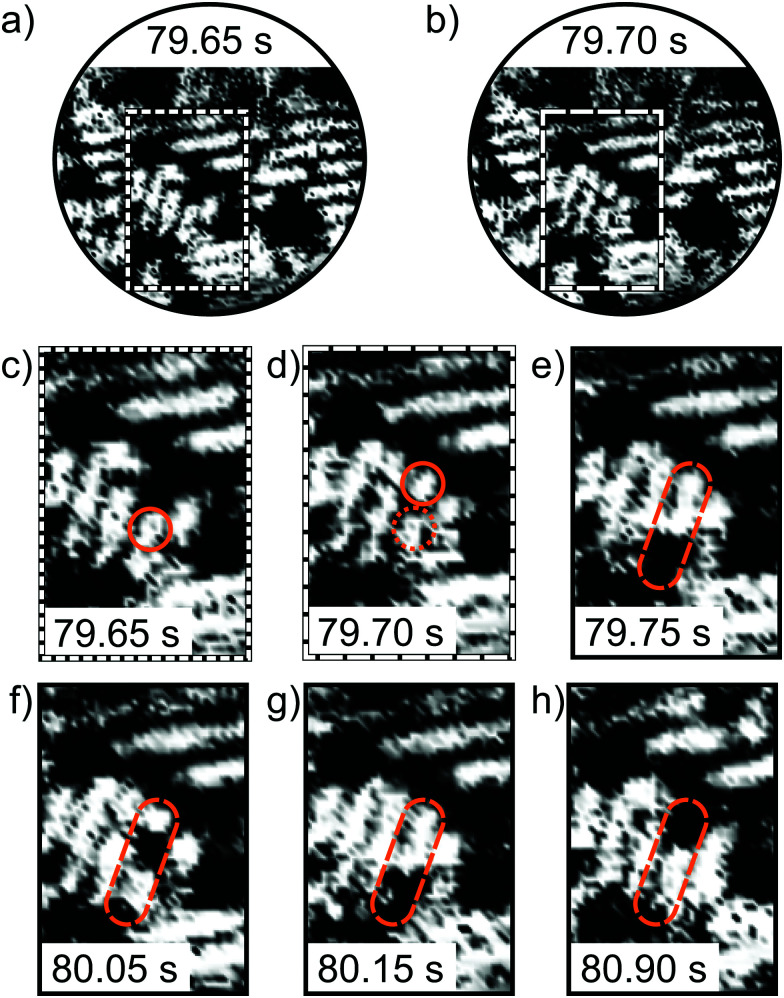
Atomically resolved individual jump events in the O(2 × 1) phase. (a) and (b) show the entire spiral scan area of 9 nm. The dashed frames indicate the region of interest, which is magnified in (c) and (d). (c)–(h) show the same region at different times as indicated by the time stamps at the bottom of each image. Hopping events of individual oxygen atoms are observed, especially in the 1D line marked in orange. Different atomic configurations are resolved. The changes in configuration are reversible. The spiral scans are extracted from the same image series as for Fig. 4 and the time stamps coincide. (*V*_S_ = 1 V, *I*_T_ = 1 nA, 20 frames s^−1^, scan dimensions of insets: 3.4 × 5.1 nm^2^).

As obvious from Fig. 4, the structural changes evolve over time and consist of consecutive dynamic events. To assess the individual dynamic events, we extracted consecutive images from the same dataset. The assigned times are synchronized with the times displayed in Fig. 4. In Fig. 5, we follow the jumps of oxygen atoms close to the boundary of two domains. For visualization the region of interest marked with dashed rectangles is magnified in Fig. 5c–h. Jump events are resolved in subsequent STM images with time differences of 50 ms (Fig. 5c–d). In the following second, the configuration in the row circled in orange changes without preferred configurations. The atoms jump back and forth. The jumps result in several atomic configurations that can also recur. The configuration of the 1D line marked in orange in Fig. 5c and h, for instance, is the same.

The dynamic processes change the appearance of the 1D lines in this single O(2 × 1) domain. However, in Fig. 5 the orientation of the 1D lines does not change over time. The question arises if changes in the orientation can also be observed on the timescale of milliseconds or if the reconstruction in Fig. 4 results from numerous undirected hopping events and thus is a process that ranges over several seconds to minutes as shown in Fig. 5.

**Fig. 6:**
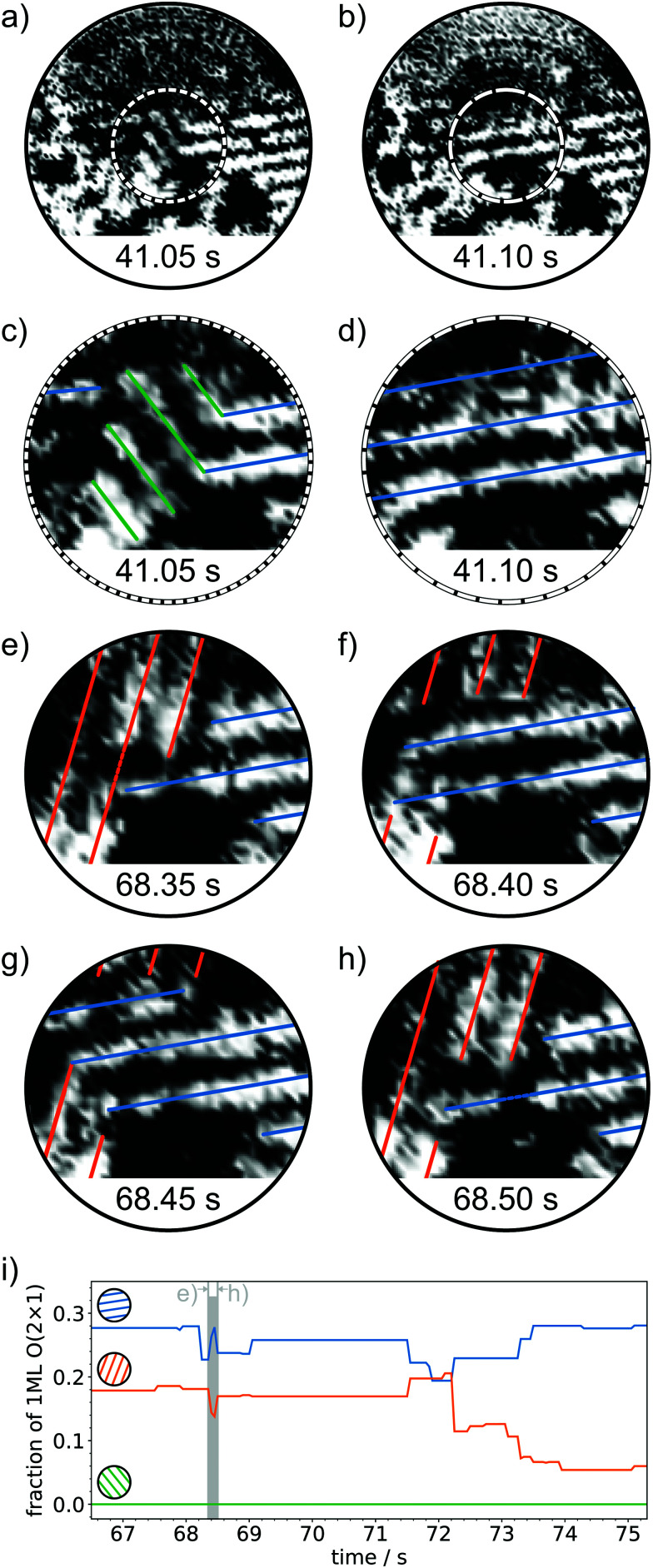
Flipping 1D line orientations in the O(2 × 1) phase. (a) and (b) show the entire spiral scan area of 9 nm. The dashed frames indicate the region of interest, which is magnified in (c) and (d). (c)–(h) show the same region at different times as indicated by the time stamps at the bottom of each image. (i) Evolution of the normalized length of 1D lines with different orientations (zoom of Fig. 4g). The time of frames (e)–(h) is marked in gray. Within short time differences of 50 to 100 ms flipping events of stripe patterns of the O(2 × 1) structure are observed. The processes are reversible. The spiral scans are extracted from the same image series as for Fig. 4 and 5. (*V*_S_ = 1 V, *I*_T_ = 1 nA, 20 frames s^−1^). The time stamps relate to Fig. 4 and 5.

To answer this question, we focus on the central region of the spiral scan, where the spatial resolution is increased due to the CAV mode of the spiral scan.^33^Fig. 6c and d show the magnified regions with color coded 1D lines. Within 50 ms, the line pattern changes abruptly and vertical, green lines “convert” to horizontal, blue lines. This phenomenon is similar to the “long-term” reorientation shown in Fig. 4 and to the theoretical flipping processes shown in Fig. 2. The flipping is captured on the millisecond timescale and fast reversible processes are observed. As shown in the upper left corner of Fig. 6e–h, the 1D line configuration changes from vertical (orange) to horizontal (blue) and *vice versa*. Similar to the atomic jump events resolved in Fig. 5, the reorientations of the 1D line profiles are reversible within very short times in the order of several 10 to 100 ms. Fig. 6i shows the curves of Fig. 4g zoomed by a factor of 10 along the time axis. The zoom illustrates the abrupt changes in the domain sizes of different orientations. The blue and the orange domain compete and they grow at the expense of the other.

This reversible competing growth is similar to the one observed on longer timescales described above. The displayed real times of fast flipping events in Fig. 6 and evolving domains in Fig. 4 agree very well. Within the time when structural changes are observed over several tens of seconds, local flip events of 1D lines in several tens of milliseconds as shown in Fig. 6c and d were observed.

It becomes obvious that the phenomena observed at the millisecond timescale directly relate to the observations over several tens of seconds or even minutes.

## 4 Conclusions

Fast structural reorientations of the 1D lines in O(2 × 1) domains on Ru(0001) were captured thanks to the increased frame rate of the spiral STM. STM measurements over several minutes with constantly high frame rates revealed the connection between fast elementary processes and long-term structural changes. DFT can explain both the high mobility in the oxygen adlayer at room temperature and the phenomenon of reorienting stripe patterns.

## Data availability statement

Real time videos, slow motion videos, and the theoretical results supporting this article have been uploaded as part of the ESI.†

## Conflicts of interest

There are no conflicts to declare.

## Supplementary Material

CP-024-D2CP02363F-s001

CP-024-D2CP02363F-s002

CP-024-D2CP02363F-s003

## References

[cit1] Binnig G., Rohrer H., Gerber C., Weibel E. (1983). Phys. Rev. Lett..

[cit2] van Hove M., Cerda J., Sautet P., Bocquet M.-L., Salmeron M. (1997). Prog. Surf. Sci..

[cit3] Kresse G., Schmid M., Napetschnig E., Shishkin M., Köhler L., Varga P. (2005). Science.

[cit4] Crommie M. F., Lutz C. P., Eigler D. M. (1993). Nature.

[cit5] Crommie M. F., Lutz C. P., Eigler D. M. (1993). Science.

[cit6] Rabe J. P., Buchholz S. (1991). Science.

[cit7] Wintterlin J., Trost J., Renisch S., Schuster R., Zambelli T., Ertl G. (1997). Surf. Sci..

[cit8] Sachs C., Hildebrand M., Völkening S., Wintterlin J., Ertl G. (2002). J. Chem. Phys..

[cit9] Marsh H. L., Deak D. S., Silly F., Kirkland A. I., Castell M. R. (2006). Nanotechnology.

[cit10] Scheiber P., Riss A., Schmid M., Varga P., Diebold U. (2010). Phys. Rev. Lett..

[cit11] Yang Y.-C., Taranovskyy A., Magnussen O. M. (2012). Langmuir.

[cit12] Yang Y.-C., Magnussen O. M. (2013). Phys. Chem. Chem. Phys..

[cit13] Henß A.-K., Sakong S., Messer P. K., Wiechers J., Schuster R., Lamb D. C., Groß A., Wintterlin J. (2019). Science.

[cit14] Wei J., Chen Y.-X., Magnussen O. M. (2020). Chem. Commun..

[cit15] Wei J., Chen Y.-X., Magnussen O. M. (2021). J. Phys. Chem. C.

[cit16] Rost M. J., Crama L., Schakel P., van Tol E., van Velzen-Williams G. B. E. M., Overgauw C. F., ter Horst H., Dekker H., Okhuijsen B., Seynen M., Vijftigschild A., Han P., Katan A. J., Schoots K., Schumm R., van Loo W., Oosterkamp T. H., Frenken J. W. M. (2005). Rev. Sci. Instrum..

[cit17] Ziegler D., Meyer T. R., Farnham R., Brune C., Bertozzi A. L., Ashby P. D. (2013). Nanotechnology.

[cit18] Madey T. E., Albert Engelhardt H., Menzel D. (1975). Surf. Sci..

[cit19] Meinel K., Wolter H., Ammer C., Beckmann A., Neddermeyer H. (1997). J. Phys.: Condens. Matter.

[cit20] Nilius N., Mitte M., Neddermeyer H. (1998). Appl. Phys. A.

[cit21] Piercy P. , Maier M. and Pfnür H., The Structure of Surfaces II, Berlin, Heidelberg, 1988, pp. 480–487

[cit22] Kresse G., Joubert D. (1999). Phys. Rev. B: Condens. Matter Mater. Phys..

[cit23] Blöchl P. E. (1994). Phys. Rev. B: Condens. Matter Mater. Phys..

[cit24] Kresse G., Furthmüller J. (1996). Comput. Mater. Sci..

[cit25] Kresse G., Furthmüller J. (1996). Phys. Rev. B: Condens. Matter Mater. Phys..

[cit26] Gura L., Yang Z., Paier J., Kalaß F., Brinker M., Junkes H., Heyde M., Freund H.-J. (2022). Phys. Rev. B.

[cit27] Monkhorst H. J., Pack J. D. (1976). Phys. Rev. B: Condens. Matter Mater. Phys..

[cit28] Methfessel M., Paxton A. T. (1989). Phys. Rev. B: Condens. Matter Mater. Phys..

[cit29] Henkelman G., Jónsson H. (1999). J. Chem. Phys..

[cit30] Heyden A., Bell A. T., Keil F. J. (2005). J. Chem. Phys..

[cit31] Sun J., Remsing R. C., Sun Z., Ruzsinszky A., Peng H., Yang Z., Paul A., Waghmare U., Wu X., Klein M. L., Perdew J. P. (2016). Nat. Chem..

[cit32] Junkes H. , Freund H.-J., Gura L., Heyde M., Marschalik P. and Yang Z., 16th International Conference on Accelerator and Large Experimental Control Systems (ICALEPCS2017), 2018, pp. 1762–1766

[cit33] Gura L., Yang Z., Brinker M., Kalaß F., Kirstaedter W., Marschalik P., Junkes H., Heyde M., Freund H.-J. (2021). Appl. Phys. Lett..

[cit34] Yang Z., Gura L., Kalaß F., Marschalik P., Brinker M., Kirstaedter W., Hartmann J., Thielsch G., Junkes H., Heyde M., Freund H.-J. (2022). Rev. Sci. Instrum..

[cit35] Over H. (2012). Chem. Rev..

[cit36] Henzler M. and Göpel W., Oberflächenphysik des Festkörpers, BG Teubner Stuttgart, 2nd edn, 1994

