# Supplementary material for: Dynamics in the O(2 × 1) adlayer on Ru(0001): bridging timescales from milliseconds to minutes by scanning tunneling microscopy
Source: Phys Chem Chem Phys. 2022 Jun 13;24(25):15265–70. doi: 10.1039/d2cp02363f (PMC9241493; doi:10.1039/d2cp02363f)
Supplement: CP-024-D2CP02363F-s003 [file CP-024-D2CP02363F-s003.pdf]

Electronic Supplementary Material (ESI) for Physical Chemistry Chemical Physics.
This journal is © the Owner Societies 2022

Supplemental Material

Dynamics in the O(2×1) Adlayer on Ru(0001): Bridging Timescales from Milliseconds to Minutes by Scanning Tunneling Microscopy

Leonard Gura,[1] Zechao Yang,[1] Joachim Paier,[2] Florian Kalaß,[1] Matthias Brinker,[1] Heinz Junkes,[1] Markus Heyde,[1,*] and Hans-Joachim Freund[1]

[1]Fritz-Haber-Institut der Max-Planck-Gesellschaft, Faradayweg 4-6, 14195 Berlin, Germany

[2]Humboldt Universität zu Berlin, Brook-Taylor-Str. 2, 12498 Berlin, Germany

E-mail: heyde@fhi-berlin.mpg.de

Contents

1. Supplementary videos 2
2. Energy differences calculated with SCAN 2

Figure S1: Top view on an O defective 3O-(4×2) adlayer of Ru(0001).......... 3

1. Supplementary videos

The supplementary videos in mp4 format show the dynamics in the O(2×1) layer on Ru(0001) as shown in Figure 3 and 6 of the main manuscript. The real time time stamps are displayed in the videos and relate to the figures in the main manuscript.

The video that relates to Figure 3, has the same image dimensions and acquisition parameters as Figure 3: V_S = 0.6 V, I_T = 1.4 nA, scan diameter = 9 nm, acquisition time = 33 ms, scan dimensions of insets: 2.7 × 2.2 nm^2. The playback speed reduced by a factor of three compared to the image acquisition rate of 30 Hz.

The video that relates to Figure 6 has the same image dimensions and acquisition parameters as Figure 6: V_S = 1 V, I_T = 1 nA, scan diameter = 9 nm, acquisition time = 50 ms, scan dimensions of insets: 3.5 × 3.5 nm^2. The playback speed reduced by a factor of ten compared to the image acquisition rate of 20 Hz.

2. Energy differences calculated with SCAN

In the main manuscript, the relevant migration pathway of oxygen and the corresponding energy differences are discussed. These values are sufficient to evaluate the jump rate and the reorientation of the stripe pattern formed by the oxygen atoms.

Here, we provide supplementary energy differences along different migration pathways that we considered in the theoretical calculations. Figure S1 provides a top view of the 3O-(4×2) unit cell on Ru(0001). Two migration pathways of oxygen are sketched with blue and orange lines. The (meta-)stable sites are labeled with A and B prefixes, respectively. The transition states (TS) are marked with black straight lines. Table S1 gives the SCAN energy differences along the longer (blue) migration pathway. Transition states are marked yellow and the relevant energy differences used in the main manuscript are underlined and bolt. Table S2 lists the SCAN energy differences along the shorter (orange) migration pathway. This pathway is found to be energetically less favorable.

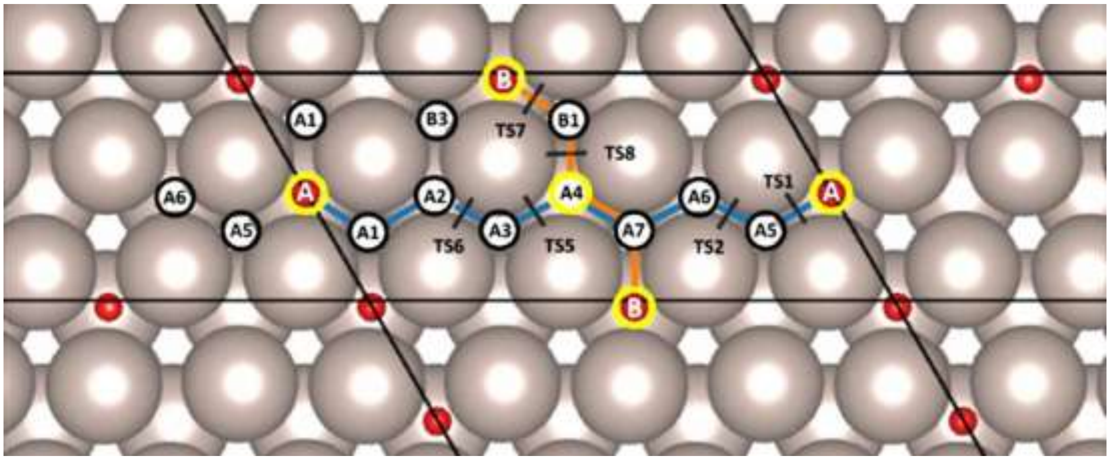

Figure S1: Top view on an O defective 3O-(4×2) adlayer of Ru(0001). The unit cell is marked with black lines. The site 'A4' refers to the O vacancy. Minimum energy structures and transition structures within a longer (blue line) and shorter migration pathway (orange line) are enumerated using the A or B prefix, respectively.

Table S1: Energy differences (eV) along the longer oxygen migration pathway marked drawn in blue in Figure S1.

Site	Label	E-SCAN (eV)	Delta (eV)	Reverse (eV)
Start	A	-1667.388	0.069	
fcc	A1	-1665.999	1.458	
hcp	A2	-1667.457	0.000	
	TS6(A2-A3)	-1666.712	0.745	0.413
fcc	A3	-1667.125	0.332	
	TS5(A4-A3)	-1666.698	0.427	0.690
hcp	A4	-1667.388	0.069	
fcc	A5	-1667.125	0.332	
	TS1 (A-A5)	-1666.700	**0.688**	0.425
hcp	A6	-1667.456	0.001	
	TS2 (A5-A6)	-1666.727	0.398	**0.729**
fcc	A7	-1666.068	1.389	

Table S2: Energy differences (eV) along the shorter oxygen migration pathway marked drawn in blue in Figure S1.

Site	Label	E-SCAN (eV)	Delta (eV)	Reverse (eV)
	TS7 (B2-B1)	-1666.450	0.938	0.267
fcc	B1	-1666.717	0.740	
	TS8 (B1-A4)	-1666.457	0.260	0.931
hcp	B2=A4	-1667.388	0.069	
fcc	B3	-1666.494	0.963	
hcp	B sits on A2	-1666.766	0.691	
